# Assessment of the capability to adopt a healthy lifestyle: insights into gender, socioeconomic factors, perceived health, and regional variations

**DOI:** 10.3389/fpubh.2025.1476401

**Published:** 2025-04-04

**Authors:** Ebtihag O. Alenzi, Wasan Ibrahim Alqahtani, Milan Adeeb Altwegri, Sadeem Mobark Alhelal, Wadha Ahmad Alyami, Danah Mohana Almohana, Reem Rashed Aldrees, Rona Shagran Alnashar, Batool Hussain Almugizel, Noura Mohammed Alshabanat, Ghada Ali Alzahrani

**Affiliations:** ^1^Family and Community Medicine Department, College of Medicine, Princess Nourah bint Abdulrahman University, Riyadh, Saudi Arabia; ^2^College of Medicine, Princess Nourah bint Abdulrahman University, Riyadh, Saudi Arabia

**Keywords:** health behavior, non-communicable diseases, sociodemographic factors, physical activity, dietary habits, health status, regional health, health perception

## Abstract

**Background:**

Maintaining a healthy lifestyle, including proper nutrition and physical activity, is essential for reducing non-communicable diseases (NCDs). However, literature lacks sufficient insight into the factors influencing individuals’ ability to adopt healthy lifestyles. Therefore, this study aimed to examine the factors affecting healthy lifestyle adoption among adults, focusing on sociodemographic aspects, regional variations, and health determinants.

**Methods:**

A cross-sectional study was conducted between February and March 2023 using convenient sampling, resulting in 999 adult participants. A validated and self-administered questionnaire, including sociodemographic data, health status, and the validated Arabic version of the Capability Assessment for Diet and Activity (CADA) scale, was distributed. Inferential statistics were reported using bi-variate analyses and multivariate regression.

**Results:**

The capability to adopt a healthy lifestyle was 3.28, with scores for physical activity and diet at 3.3 and 3.27, respectively. Bivariate analyses revealed significant associations of age, educational level, income, housing type, region, weight, and perceived physical and psychological health status with the capability to adopt a healthy lifestyle. In adjusted analyses, males had lower diet scores (*β* = −0.36, *p* = 0.026) than females. Participants with incomes below 7,000 SR had lower total CADA scores (*β* = −0.36, *p* = 0.064) and lower physical activity scores (*β* = −0.43, *p* = 0.026) than those earning >25,000 SR. Participants residing in family houses or duplexes had higher total CADA scores than those in smaller properties. Participants in central regions had significantly higher scores for adopting healthy lifestyles (*β* = 0.46, *p* = 0.040) than those in other areas. Overweight had higher total CADA scores (*β* = 0.58, *p* = 0.011) and healthier diet scores (*β* = 0.64, *p* < 0.01) than extremely obese. Furthermore, positive perceptions of physical and/or mental health were linked to higher scores in adopting healthy lifestyles.

**Conclusion:**

The findings underscore the impact of gender, income, housing type, region, and perceived health status on individuals’ ability to engage in physical activity and adopt a healthy diet. Thus, health strategies that address these differences could enhance the adoption of healthier lifestyles and reduce the prevalence of lifestyle-related diseases in the population.

## Background

Maintaining a healthy lifestyle with proper nutrition and regular physical activity is essential for short- and long-term health benefits (1). Physical activity has numerous physical and mental health benefits, including increased cardiorespiratory and muscular fitness, improved cognition, and a reduced risk of depression and/or anxiety (2).

Globally, inadequate physical activity and unhealthy dietary habits are major contributors to the rising prevalence of non-communicable diseases (NCDs) (3). On a regional level, the Kingdom of Saudi Arabia (KSA) has faced similar health challenges. Studies indicate that physical inactivity among Saudi adults has varied between 4 and 44.5% over the years (4). The most recent data from a 2020 population-based study shows a national obesity prevalence of 24.7%, reflecting a decline compared to previous years (25.6% in 2018 and 28.7% in 2013) (5–7). Although the weighted obesity prevalence is declining (6, 7), national efforts continue to emphasize the importance of adopting healthy lifestyle practices to combat obesity and related health conditions.

In response to these health challenges, the Kingdom of Saudi Arabia (KSA) has launched numerous national health initiatives aligned with Vision 2030 to improve public health and well-being (8–12). These programs address public health challenges and increase physical activity, healthy eating habits, and overall community wellness. However, there is a gap in the literature regarding a comprehensive assessment of individuals’ capability to adopt healthy lifestyles and its related factors, which are crucial for informing public health policies and interventions tailored to the population’s needs.

Many factors may affect dietary practices and physical activity levels, including sociodemographic status, education levels, psychological factors, and environmental factors (13, 14). In Saudi Arabia, rapid urbanization and sedentary work environments may lead to challenges in maintaining healthy lifestyles (15). Understanding the factors influencing these capabilities can provide insights into disparities across the kingdom’s demographic groups, geographic regions, and socioeconomic strata.

The present study aimed to fill this gap by examining the capability of Saudi adults aged 18 years and above to adopt healthy lifestyles. Using a population-based cross-sectional approach, this research investigated the effects of various factors, including socioeconomic status, demographic characteristics, regional variations, and health status, on individuals’ ability to engage in regular physical activity and maintain a nutritious diet.

The conceptual behavioral framework used for this study is the Capability-Opportunity-Motivation-Behavior (COM-B) model (16). Generally, capability refers to a person’s psychological and physical ability to adopt certain behaviors or to engage in certain activities (17). According to the COM-B model, adopting healthy behavior, such as engaging in physical activity or maintaining a nutritious diet, could be impacted by three main elements: capability, opportunity, and motivation.

## Methods

### Study design and sampling

A cross-sectional observational study using a convenient sampling technique was conducted among adults to identify the factors affecting the adoption of a healthy lifestyle. Using a convenient sampling technique with no gender restrictions, the study included 999 participants. In this study, demographic factors, socioeconomic status, and health determinants were considered independent variables, while being 18 years old and willing to participate were the inclusion criteria.

### Procedures

The study was approved by the Institutional Review Board (IRB) at Princess Nourah bint Abdulrahman University (PNU), Riyadh, Saudi Arabia (IRB log number: 23–0176). Data was collected using an online questionnaire distributed across regions of Saudi Arabia through social media platforms, including WhatsApp, Twitter, Telegram, Snapchat, and Facebook. The recruitment process was voluntary, and participants were fully informed about the study objectives, their right to withdraw at any time, and the anonymous nature of their participation. Informed consent was obtained from all eligible participants who agreed to participate. A total of 999 participants completed the online questionnaire during the data collection period from February to March 2023. This large sample size resulted from the broad questionnaire distribution across multiple social media platforms, ensuring a diverse participant pool. The data collected was reliable and representative of the broader population.

### Measures

#### Sociodemographic characteristics

Sociodemographic data were collected using a structured questionnaire, which included various factors such as sex, nationality, age, marital status, education level, occupation, family monthly income, housing type, and region of residence. Specifically, participants were asked to report their sex (male or female), nationality (Saudi or non-Saudi), and age (in years). Age was then categorized into the following groups based on the sample distribution: 18–24 years, 25–44 years, 45–54 years, and 55 years and older. Marital status was categorized as either single or married. Participants were also asked about their education level (pre-undergraduate, undergraduate, or higher education), occupation (housewife, a student in a health major, a student in a non-health major, unemployed, an employee in the health field), and monthly family income (less than 7,000 Saudi riyals, from 7,000 to 15,000 Saudi riyals, from 15,000 to 25,000 Saudi riyals, more than 25,000 Saudi riyals). Housing type was classified as family house, duplex, apartments, and others. Also, the region of residence was categorized into Central, Southern, Northern, Western, and Eastern regions based on the participants’ place of residence.

#### Health status

Health status data included several measures, such as weight status categories, perceived physical health, perceived psychological/mental health status, the presence of any chronic physical conditions, and the presence of any chronic psychological diseases. For weight status, participants were asked to report their height in centimeters and weight in kilograms. Body mass index (BMI) was calculated using the standard formula (weight in kilograms divided by height in meters squared). Based on the calculated BMI, participants were categorized as underweight (BMI <18.5), normal weight (BMI 18.5–24.9), overweight (BMI 25–29.9), obese (BMI 30–34.9), and extremely obese (BMI ≥ 35) (18). For perceived physical health, participants were asked if they suffer from any physical problem that limits their activity and ability to perform their daily tasks. Likewise, for perceived psychological health, participants were asked if they suffer from any psychological problem that limits their activity and ability to perform their daily tasks. Chronic conditions were determined through self-report, where participants indicated whether they had been diagnosed with any chronic physical or psychological diseases.

#### Healthy lifestyle capability

The outcome variable was the individuals’ capability to adopt a healthy lifestyle, which was measured using the validated Arabic version of the Capability Assessment for Diet and Activity (CADA) scale (19). This scale, developed by Ferrer et al., assesses individuals’ opportunities to engage in healthy behaviors, such as maintaining a healthy diet and participating in physical activity (20). The CADA scale consists of two main components: diet (14 items) and physical activity (11 items). For the diet component, there are four subscales: diet opportunity (5 items), diet barriers (3 items), diet knowledge (3 items), and diet time (3 items). The physical activity component includes subscales for physical activity convenience (3 items), neighborhood factors (5 items), and physical activity barriers (3 items). The scale uses a 5-point Likert scale, ranging from “strongly disagree” to “strongly agree.” The Arabic version of the CADA scale was translated and validated within the Arabic context by Alhaji et al., with an excellent content validity index, showing 99.3% representativeness of the domain and 98.6% clarity (19).

### Statistical analysis

Frequencies and percentages were reported for sociodemographic and health status variables. Descriptive statistics, including means and standard deviations (SDs), were calculated for the total CADA, diet, and physical activity scores. *T*-tests were used to examine the bivariate associations between sociodemographic and health status variables with two categories (e.g., sex, marital status) and the total CADA, diet, and physical activity scores. One-way analysis of variance (ANOVA) was used to assess associations between sociodemographic and health status variables with more than two categories (e.g., age, educational level) and the total CADA, diet, and physical activity scores.

Multivariate analyses were conducted using ordinary least squares (OLS) regression to examine the relationship between sociodemographic variables, health status, and lifestyle factors with the total CADA, diet, and physical activity scores. A *p*-value of less than 0.05 was considered statistically significant for all analyses. All statistical analyses were performed using IBM SPSS Statistics.

## Results

### Main characteristics of the sample

The attributes of the study sample are demonstrated in Table 1. The study sample consisted of 999 individuals, 35.7% of whom were males and 64.3% of whom were females. The majority of participants (95.4%) were Saudi nationals, and the largest group was aged 18–24 years (36.3%), followed by those aged 25–44 years (33.3%). A slight majority of married individuals (55.3%) in the marital status group compared to non-married (44.7%). For educational level, the majority were undergraduates (65.8%), with significant proportions at the pre-undergraduate (19.0%) and postgraduate levels (15.2%). Occupationally, the sample exhibited diverse statuses: 36.8% were employed in non-health fields, 15.1% were unemployed, and notable proportions were students in both health (16.0%) and non-health majors (14.1%). For family monthly income, there was a balanced distribution across four groups: less than 7,000 Saudi riyals (23.2%), from 7,000 to 15,000 Saudi riyals (30.1%), from 15,000 to 25,000 Saudi riyals (24.1%), and more than 25,000 Saudi riyals (22.5%). Housing types were predominantly family houses (67.1%), with 19% living in apartments (19.0%) and 7% living in duplexes. Geographically, the central region of Saudi Arabia had the highest residential representation (72.6%), with 8 to 9% of the participants residing in southern and eastern regions. The weight status distribution indicated significant proportions in the normal weight (34.4%) and overweight (29.7%) categories, with fewer individuals classified as underweight (7.2%), obese (15.8%), or extremely obese (7.9%). Regarding diagnosed chronic illnesses, 20.5% of participants reported a chronic physical illness, while 6.7% reported a chronic mental illness. In terms of daily activity limitations, 13.8% of respondents indicated physical issues, while 14.4% reported psychological challenges.

**Table 1:** Characteristics of the study sample (*n* = 999).

Variables	Frequency	Percent (%)
Gender		
Male	357	35.7
Female	642	64.3
Nationality		
Saudi	953	95.4
Non-Saudi	46	4.6
Age (years)		
18–24	363	36.3
25–44	333	33.3
45–54	184	18.4
More than or equal 55	119	11.9
Marital status		
Non-married	447	44.7
Married	552	55.3
Educational level		
Pre-undergraduate	190	19.0
Undergraduate	657	65.8
Postgraduate	152	15.2
Occupation		
Housewife	136	13.6
A student in a health major	160	16.0
A student in a non-health major	141	14.1
Unemployed	151	15.1
An employee in the health field	43	4.3
An employee in a non-health field	368	36.8
Family monthly income		
Less than 7,000 Saudi riyals	232	23.2
From 7,000 to 15,000 Saudi riyals	301	30.1
From 15,000 to 25,000 Saudi riyals	241	24.1
More than 25,000 Saudi riyals	225	22.5
What type of housing do you live in?		
Family house	670	67.1
Duplex	70	7.0
Apartments	190	19.0
Other	69	6.9
In which region of the KSA do you live?		
Central region	725	72.6
Southern region	91	9.1
Northern region	14	1.4
Western region	88	8.8
Eastern region	81	8.1
Weight status category		
Underweight	72	7.2
Normal weight	344	34.4
Overweight	297	29.7
Obese	158	15.8
Extremely obese	79	7.9
Perceived physical problems that limit daily activities:		
Yes	138	13.8
No	861	86.2
Perceived psychological problems that limit daily activities:		
Yes	144	14.4
No	855	85.6
Presence of any diagnosed chronic physical illness:		
Yes	205	20.5
No	794	79.5
Presence of any diagnosed chronic mental illness:		
Yes	67	6.7
No	932	93.3

### Descriptive analysis of capability for diet and physical activity

Table 2 summarizes the descriptive statistics of capability assessment for diet and activity among participants. The total CADA score averaged 3.28 (± 0.48) out of 5. The average diet CADA score was 3.27 (± 0.46), with various component scores: diet opportunity (items 1–5) averaged at 3.28 (± 0.68), diet barriers (items 6–8) averaged at 2.67 (± 0.86), diet knowledge (items 9–11) averaged at 3.28 (± 1.06), and diet duration (items 12–14) averaged at 3.25 (± 0.92). The average physical activity CADA score was 3.30 (± 0.67). The component scores for physical activity showed variability: physical activity convenience (items 15–17) averaged 2.98 (± 0.96), neighborhood factors (items 18–22) averaged 3.17 (± 0.96), and physical activity barriers (items 23–25) averaged 2.18 (± 1.03).

**Table 2:** Descriptive statistics of capability assessment for diet and activity (CADA) (*n* = 999).

Variables	Mean	Std. Deviation
1. Total CADA score	3.28	0.48
2. Diet CADA score	3.27	0.46
2.1 Diet opportunity (items 1–5)	3.28	0.68
2.2 Diet barriers (items 6–8)	2.67	0.86
2.3 Diet knowledge (items 9–11)	3.28	1.06
2.4 Diet time (items 12–14)	3.25	0.92
3. Physical Activity CADA score	3.30	0.67
3.1 Physical activity convenience (items 15–17)	2.98	0.96
3.2 Neighborhood (items 18–22)	3.17	0.96
3.3 Physical activity barriers (items23-25)	2.18	1.03

### Bivariate analysis of factors related to capability for diet and physical activity

The associations of socioeconomic and demographic factors, in addition to health status, with individuals’ capability to adopt healthy lifestyle behaviors were presented in Table 3.

**Table 3:** Bivariate associations of sociodemographic factors and health status with people’s capability for adopting healthy lifestyle behaviors (*n* = 999).

Variables	Overall, CADA score		Overall physical activity score		Overall diet score	
	X ± SD	*p*-value	X ± SD	*p*-value	X ± SD	*p*-value
Socioeconomic data						
Gender						
Males	3.28 ± 0.48	0.920 ^a^	3.27 ± 0.66	0.157 ^a^	3.29 ± 0.46	0.147 ^a^
Females	3.29 ± 0.48		3.34 ± 0.68		3.25 ± 0.44	
Nationality						
Saudi	3.28 ± 0.48	0.261 ^a^	3.30 ± 0.69	0.272 ^a^	3.28 ± 0.46	0.406 ^a^
Non-Saudi	3.20 ± 0.51		3.19 ± 0.86		3.22 ± 0.48	
Age						
18–24	3.29 ± 0.46	<0.01 ^b *^	3.31 ± 0.65	<0.01 ^b *^	3.28 ± 0.45	<0.01 ^b *^
25–44	3.20 ± 0.49		3.19 ± 0.67		3.20 ± 0.48	
45–54	3.37 ± 0.48		3.42 ± 0.70		3.32 ± 0.43	
More than or equal 55	3.36 ± 0.45		3.36 ± 0.63		3.36 ± 0.41	
Marital status						
Non-married	3.28 ± 0.46	0.739 ^a^	3.29 ± 0.66	0.590 ^a^	3.27 ± 0.46	0.999 ^a^
Married	3.29 ± 0.49		3.31 ± 0.67		3.27 ± 0.46	
Educational level						
Pre-undergraduate	3.21 ± 0.50	<0.01 ^b *^	3.20 ± 0.69	<0.01 ^b *^	3.22 ± 0.49	0.079 ^b^
Undergraduate	3.28 ± 0.48		3.29 ± 0.67		3.27 ± 0.45	
Postgraduate	3.38 ± 0.44		3.43 ± 0.65		3.34 ± 0.42	
Occupation						
Housewife	3.23 ± 0.49	0.547 ^b^	3.18 ± 0.63	0.212 ^b^	3.27 ± 0.48	0.775 ^b^
A student in health major	3.32 ± 0.46		3.37 ± 0.64		3.28 ± 0.45	
A student in a non-health major	3.24 ± 0.44		3.25 ± 0.63		3.24 ± 0.44	
Unemployed	3.31 ± 0.48		3.29 ± 0.68		3.32 ± 0.45	
An employee in the health field	3.31 ± 0.48		3.33 ± 0.75		3.30 ± 0.44	
An employee in a non-health field	3.29 ± 0.50		3.32 ± 0.70		3.26 ± 046	
Family monthly income						
< 7,000 Saudi riyals	3.21 ± 0.53	<0.01 ^b *^	3.18 ± 0.71	<0.01 ^b *^	3.23 ± 0.49	0.052 ^b^
7,000 to 15,000 Saudi riyals	3.23 ± 0.47		3.21 ± 0.65		3.24 ± 045	
15,000 to 25,000 Saudi riyals	3.32 ± 0.44		3.35 ± 0.60		3.30 ± 0.46	
> 25,000 Saudi riyals	3.28 ± 0.48		3.47 ± 0.69		3.33 ± 0.42	
Type of housing						
Family House	3.32 ± 0.48	<0.01 ^b *^	3.35 ± 0.68	<0.01 ^b *^	3.31 ± 0.46	<0.01 ^b *^
Duplex	3.32 ± 0.41		3.30 ± 0.53		3.34 ± 0.46	
Apartments	3.20 ± 0.46		3.22 ± 0.64		3.18 ± 0.43	
Other	3.09 ± 0.48		3.02 ± 0.64		3.14 ± 0.45	
Region						
Central region	3.31 ± 0.47	<0.01 ^b *^	3.33 ± 0.67	0.024 ^b *^	3.30 ± 0.46	<0.01 ^b *^
Southern region	3.11 ± 0.46		3.10 ± 0.66		3.12 ± 0.38	
Northern region	3.16 ± 0.39		3.31 ± 0.66		3.05 ± 0.28	
Western region	3.26 ± 0.433		3.24 ± 0.62		3.28 ± 0.43	
Eastern region	3.25 ± 0.55		3.24 ± 0.72		3.27 ± 0.49	
Weight status category						
Underweight	3.32 ± 0.43	<0.01 ^b *^	3.37 ± 0.65	<0.01 ^b *^	3.27 ± 0.433	0.011 ^b *^
Normal weight	3.29 ± 0.50		3.30 ± 0.70		3.28 ± 0.48	
Overweight	3.34 ± 0.47		3.38 ± 0.67		3.30 ± 0.45	
Obese	3.22 ± 0.47		3.18 ± 0.66		3.26 ± 0.46	
Extremely obese	3.12 ± 0.43		3.14 ± 0.62		3.10 ± 0.44	
Health status						
Perceived physical problem						
Yes	3.08 ± 0.45	<0.01 ^a *^	2.99 ± 0.60	<0.01 ^a *^	3.15 ± 0.43	<0.01 ^a *^
No	3.32 ± 0.48		3.34 ± 0.67		3.29 ± 0.46	
Perceived psychological problem						
Yes	3.11 ± 0.45	<0.01 ^a *^	3.07 ± 0.64	<0.01 ^a *^	3.14 ± 0.43	<0.01 ^a *^
No	3.31 ± 0.48		3.33 ± 0.67		3.30 ± 0.46	
Diagnosed physical problem						
Yes	3.25 ± 0.48	0.280 ^a^	3.22 ± 0.65	0.083 ^a^	3.27 ± 0.43	0.989 ^a^
No	3.29 ± 0.48		3.31 ± 0.68		3.27 ± 0.46	
Diagnosed psychological problem						
Yes	3.23 ± 0.39	0.306 ^a^	3.16 ± 0.58	0.077 ^a^	3.28 ± 0.41	0.899 ^a^
No	3.29 ± 0.48		3.31 ± 0.68		3.27 ± 0.46	

a, *T*-test; b, One-way analysis of variance (ANOVA); * = *p* < 0.05 was considered significant.

Regarding overall CADA scores, there were significant associations with age, education level, family income, type of housing, region of residence, weight status, perceived physical health status, and perceived psychological health status. Compared with individuals in other age groups, individuals aged 45 to 54 years had the highest scores (3.37 ± 0.48). For educational level, participants who had postgraduate degrees had higher overall CADA (3.38 ± 0.44) scores than did those with other educational levels. Significant differences were observed in family monthly income: participants with higher incomes (15,000 to 25,000 Saudi riyals and > 25,000 Saudi riyals) and/or living in family houses or duplexes had the highest scores for total CADA scores. Participants from the central region scored higher in overall CADA (3.31 ± 0.47) than did those from other regions. Participants classified as overweight exhibited higher overall CADA (3.34 ± 0.47) than did those in other categories. Individuals who perceived that they had no physical problems that could limit their daily activities had higher overall CADA (3.32 ± 0.48) scores than did their counterparts. Similarly, participants who perceived that they had no psychological problems that could limit their daily activities had higher overall CADA (3.31 ± 0.48) scores than did those who perceived that they had psychological problems.

Likewise of overall CADA scores, scores of physical activities were significantly associated with age, education level, family income, type of housing, region of residence, weight status, perceived physical health status, and perceived psychological health status. Individuals aged 45 to 54 years had the highest scores for physical activity (3.42 ± 0.70) as compared with individuals in other age groups. Also, participants who had postgraduate degrees had higher physical activity (3.43 ± 0.65) scores than did those with other educational levels. Individuals with higher family incomes (15,000 to 25,000 Saudi riyals and > 25,000 Saudi riyals) and those who live in family houses or duplexes had the highest scores for physical activity too. People resided in central region scored higher in physical activity (3.33 ± 0.67) as compared to other regions. Participants who were categorized as overweight had higher scores of physical activities (3.38 ± 0.67) than did other groups. Individuals who perceived that they had positive health status had higher physical activity (3.34 ± 0.67) scores than did their counterparts. In addition, participants who think that they had no psychological health issues had higher physical activity (3.33 ± 0.67) than did those who perceived that they had psychological problems.

However, diet scores were only significantly associated with age, type of housing, region of residence, weight status, perceived physical health status, and perceived psychological health status. Participants aged older than 54 years had the highest score for diet (3.36 ± 0.41) compared to other age groups. Further, participants who live in family houses or duplexes and residents of central region scored higher in diet (3.30 ± 0.46) than others. Participants who were overweight showed higher diet (3.30 ± 0.45) scores than did those in other weight groups. Also, people who perceived that they had no physical health issues reported higher diet (3.29 ± 0.46) scores than did their comparators. Participants who belief that they had no psychological health issues had higher diet (3.30 ± 0.46) scores than did those who belief that they had problems.

### Multivariate analysis of factors related to capability for diet and physical activity

Table 4 demonstrated the adjusted associations of sociodemographic factors and health status, with people’s overall capability to adopt healthy lifestyle behaviors and their capability for Diet and Physical Activity. The table outlines the statistical significance (*p*-value) and the *β* coefficients with their corresponding 95% confidence intervals (CIs) for various variables across three main scores: overall CADA, physical activity, and diet.

**Table 4:** The adjusted association of sociodemographic factors and health status with people’s capability for adopting healthy lifestyle behaviors (*n* = 999).

Variables	Overall, CADA Score		Overall Physical Activity Score		Overall Diet Score	
	*β* (95% CI)	*p*-value	*β* (95% CI)	*p*-value	*β* (95% CI)	*p*-value
Socioeconomic data						
Gender						
Males	−0.21 (−0.51, 0.10)	0.183	0.60 (−0.25, 0.36)	0.705	−0.36 (−0.67, −0.06)	0.020 *
Females	Reference group					
Nationality						
Saudi	−0.12 (−0.68, 0.43)	0.661	−0.01 (−0.56, 0.54)	0.970	−0.11 (−0.66, 0.44)	0.695
Non-Saudi	Reference group					
Age						
18–24	0.10 (−0.55, 0.75)	0.753	0.70 (−0.59, 0.72)	0.844	0.20 (−0.45, 0.85)	0.554
25–44	−0.38 (−0.85, 0.08)	0.106	−0.26 (−0.32, 0.21)	0.281	−0.35 (−0.82, 0.12)	0.139
45–54	0.18 (−0.30, 0.65)	0.461	0.29 (−0.19, 0.76)	0.233	0.01 (−0.46, 0.48)	0.967
More than or equal 55	Reference group					
Marital status						
Non-married	−0.07 (−0.48, 0.34)	0.736	−0.12 (−0.53, 0.29)	0.560	0.00 (−0.41, 0.41)	0.998
Married	Reference group					
Educational level						
Pre-undergraduate	−0.35 (−0.81, 0.10)	0.129	−0.24 (−0.70, 0.22)	0.300	−0.33 (−0.79, 0.13)	0.159
Undergraduate	0.48 (−0.51, 0.24)	0.478	−0.01 (−0.39, 0.36)	0.944	−0.19 (−0.56, 0.18)	0.320
Postgraduate	Reference group					
Occupation						
Housewife	−0.14 (−0.44, 0.41)	0.949	−0.01 (−0.44, 0.42)	0.952	0.06 (−0.37, 0.49)	0.780
A student in health major	−0.04 (−0.56, 0.48)	0.882	0.23 (−0.29, 0.76)	0.385	−0.32 (−0.85, 0.20)	0.224
A student in a non-health major	−0.35 (−0.86, 0.16)	0.181	−0.10 (−0.61, 0.41)	0.695	−0.41 (−0.92, 0.10)	0.118
Unemployed	0.13 (−0.28, 0.53)	0.540	0.04 (−0.36, 0.44)	0.850	0.14 (−0.26, 0.54)	0.499
An employee in the health field	0.18 (−0.40, 0.67)	0.541	0.19 (−0.39, 0.77)	0.526	0.04 (−0.54, 0.62)	0.898
An employee in a non-health field	Reference group					
Family monthly income						
< 7,000 Saudi riyals	−0.36 (−0.73, 0.02)	0.064	−0.43 (−0.81, −0.05)	0.026 *	−0.21 (−0.58, 0.17)	0.283
7,000 to 15,000 Saudi riyals	−0.36 (−0.70, −0.02)	0.036 *	−0.43 (−0.77, −0.09)	0.014 *	−0.14 (−0.47, 0.20)	0.427
15,000 to 25,000 Saudi riyals	−0.14 (−0.45, 0.20)	0.419	−0.23 (−0.56, 0.11)	0.185	−0.07 (−0.41, 0.27)	0.688
> 25,000 Saudi riyals	Reference group					
Type of housing						
Family House	0.67 (0.19, 1.13)	<0.01 *	0.65 (0.18, 1.12)	<0.01 *	0.44 (−0.03, 0.91)	0.064
Duplex	0.80 (0.19, 1.41)	<0.01 *	0.73 (0.12, 1.34)	0.019 *	0.62 (0.01, 1.23)	0.045 *
Apartments	0.44 (−0.06, 0.49)	0.085	0.52 (0.02, 1.02)	0.042 *	0.15 (−0.35, 0.65)	0.557
Other	Reference group					
Region						
Central region	0.45 (0.02, 0.88)	0.040 *	0.37 (−0.06, 0.80)	0.087	0.29 (−0.14, 0.72)	0.187
Southern region	−0.28 (−0.83, 0.28)	0.329	−0.26 (−0.82, 0.29)	0.351	−0.31 (−0.86, 0.24)	0.273
Northern region	−0.15 (−1.19, 0.90)	0.779	0.25 (−0.79, 1.30)	0.633	−0.61 (−1.65, 0.44)	0.257
Western region	0.26 (−0.24, 0.89)	0.263	0.13 (−0.44, 0.69)	0.675	0.33 (−0.24, 0.90)	0.253
Eastern region	Reference group					
Weight status category						
Underweight	0.52 (−0.09, 1.13)	0.095	0.35 (−0.27, 0.96)	0.268	0.46 (−0.15, 1.07)	0.142
Normal weight	0.35 (−0.11, 0.81)	0.142	0.10 (−0.36, 0.57)	0.666	0.42 (−0.05, 0.88)	0.079
Overweight	0.58 (0.13, 1.03)	0.011 *	0.31 (−0.14, 0.76)	0180	0.64 (0.19, 1.09)	<0.01 *
Obese	0.12 (−0.36, 0.60)	0.642	−0.23 (−0.71, 0.25)	0.347	0.40 (−0.08, 0.89)	0.102
Extremely obese	Reference group					
Health status						
Perceived physical problem						
No	0.66 (0.30, 1.00)	<0.01 *	0.69 (0.33, 1.04)	<0.01 *	0.42 (0.07, 0.77)	0.019 *
Yes	Reference group					
Perceived psychological problem						
No	0.70 (0.33, 1.00)	<0.01 *	0.55 (0.18, 0.91)	<0.01 *	0.66 (0.30, 1.03)	<0.01 *
Yes	Reference group					
Diagnosed physical problem						
No	0.01 (−0.30, 0.32)	0.942	0.03 (−0.28, 0.34)	0.853	−0.06 (−0.37, 0.25)	0.695
Yes	Reference group					
Diagnosed psychological problem						
No	−0.28 (−0.75, 0.20)	0.251	−0.02 (−0.50, 0.46)	0.932	−0.41 (−0.89, 0.07)	0.092
Yes	Reference group					

95% CI, 95% confidence intervals; *, *p* < 0.05 was considered significant.

Overall, gender, family income, housing type, region, weight status, and perceived health status were significantly associated with the capability to adopt a healthy lifestyle. On the other hand, age and education level groups did not significantly differ in their overall CADA, physical activity, or diet scores. Family monthly income demonstrated a significant association with overall CADA as individuals with lower incomes (< 7,000 Saudi riyals and 7,000 to 15,000 Saudi riyals) had lower overall CADA scores than those with higher incomes (> 25,000 Saudi riyals). Individuals who lived in family houses and duplexes had higher overall CADA scores than those who lived in apartments and other housing types. Participants who resided in central regions were significantly more capable of adopting healthy lifestyles than those who resided in other regions (*β* = 0.456, 95% CI: 0.02 to 0.88). Additionally, overweight individuals had higher overall CADA (*β* = 0.58, 95% CI: 0.13 to 1.03) scores than extremely obese individuals. In contrast, individuals who were underweight or had normal weight did not significantly differ from extremely obese individuals in terms of their capabilities to adopt a healthy lifestyle. Similarly, individuals without perceived physical problems exhibited higher overall CADA (*β* = 0.66, 95% CI: 0.30 to 1.00) scores than those who reported having such problems. Moreover, individuals without perceived psychological problems also had higher overall CADA scores (*β* = 0.70, 95% CI: 0.33 to 1.00) than their counterparts with psychological concerns. Like the unadjusted results, diagnosed physical or psychological problems were not significantly associated with the lifestyle behavior scores.

For physical activity, family income, housing type, perceived physical health, and perceived psychological health were independently associated with physical activity. Participants with lower incomes had lower scores than those with higher incomes (> 25,000 Saudi riyals). Individuals who lived in independent or big houses, such as family houses, had higher physical activity scores than those in smaller housing properties. People without perceived physical problems reported higher scores for physical activity (*β* = 0.69, 95% CI: 0.33 to 1.04) than those who reported perceived physical health issues. Also, participants who perceived that they had no psychological problems that could limit their daily activities had higher physical activity scores (*β* = 0.55, 95% CI: 0.18 to 0.91) than those who perceived they had psychological issues.

For adopting a healthy diet, gender, weight status, perceived physical health, and perceived psychological health were significantly associated with diet scores. Compared with females, males exhibited a lower diet score (*β* = −0.36, 95% CI: −0.67 to −0.06). Also, overweight individuals had higher diet (*β* = 0.64, 95% CI: 0.19 to 1.09) scores than did extremely obese individuals. Similarly, individuals without perceived physical problems reported higher scores for diet (*β* = 0.42, 95% CI: 0.07 to 0.77) than did those who reported having such problems. Likewise, individuals without perceived psychological problems also had higher diet (*β* = 0.66, 95% CI: 0.30 to 1.03) scores than their counterparts with psychological concerns.

## Discussion

The study aimed to assess the Saudi population’s capability to adopt healthy lifestyles, including physical activity and diet. The sample was diverse in terms of gender, nationality, and age, which allowed for a comprehensive evaluation of lifestyle adoption across different demographic groups.

In the context of healthy lifestyle adoption, our findings indicated generally high capability levels in the Saudi population to adopt healthy lifestyles (3.28 ± 0.48), with a slightly greater physical activity capacity than diet (3.30 vs. 3.27). Additionally, the component scores ranged from 2.18 to 3.28, reflecting the variability in participants’ perceived capabilities in managing their diet and physical activity and highlighting strengths and areas for potential improvement in lifestyle management. These results suggest a promising baseline for lifestyle adoption but also emphasize the need for targeted interventions to address specific areas of improvement. The associations of these capabilities to adopt healthy lifestyles (both diet and physical) with socioeconomic, demographic, and health status factors were assessed to identify these areas for improvement.

The findings highlight several key determinants influencing individuals’ ability to adopt healthy lifestyles.

In the bivariate analyses, significant associations were found between age, education level, family income, type of housing, region of residence, weight status, perceived physical health status, perceived psychological health status, and individuals’ capability to adopt a healthy lifestyle. Age emerged as a significant factor, with middle-aged participants (45–54 years) showing greater engagement in overall CADA and physical activity. In comparison, older individuals (older than 54) were more attentive to adopting a healthy diet. Educational attainment, particularly postgraduate degrees, was associated with better overall CADA and physical activity scores, underscoring the role of education in promoting healthier lifestyles. Additionally, higher family incomes were associated with enhanced overall CADA and physical activity levels, indicating that financial stability may contribute to better engagement in these health-related behaviors. Housing type and region of residence also played crucial roles, with residents of family houses or duplexes and those living in the central region exhibiting higher overall CADA, physical activity, and diet scores. Weight status was strongly associated with healthier behaviors, as overweight participants consistently reported greater engagement in overall CADA, physical activity, and diet. Moreover, perceived physical and psychological health status significantly influenced the capabilities to adopt healthy lifestyles, emphasizing the importance of mental and physical well-being in promoting healthy behaviors. Individuals without perceived physical or psychological limitations in daily activities scored higher in overall CADA, physical activity, and diet than their counterparts. This could suggest emphasizing the importance of mental and physical well-being in promoting healthy behaviors. However, the presence of chronic diseases was not significantly associated with the capability to adopt healthy lifestyles.

The multivariate analysis conducted in this study explored the complex interplay of demographic factors, socioeconomic factors, and health status in relation to individuals’ capability to adopt healthy lifestyle behaviors. The findings underscore the robustness of the suggested associations of gender, family income, housing type, region of residence, weight status, and perceived health status with individuals’ capability to adopt healthy lifestyles. The adjusted results indicated that these factors could influence people’s health behaviors independently. At the same time, age and education could indirectly affect the ability to adopt a healthy lifestyle, as they could mediate the impact of other factors, such as family income. This finding is consistent with previous studies suggesting that age and education indirectly affect healthy lifestyles by mediating the impact of factors such as family income or environmental factors, illustrating the complex interplay of socioeconomic status, health behaviors, and health outcomes (21, 22). Gender emerged as a noteworthy factor influencing dietary habits, with males exhibiting lower diet scores than females. In line with our findings, a prior study conducted in the UK revealed that men’s limited knowledge related to diet and nutrition accounted for a significant portion of their lower intake of fruits and vegetables (23). This finding suggests potential gender-specific differences in dietary preferences or adherence to healthy eating habits, warranting further investigation into nutrition-related knowledge, dietary norms, and social roles. Family income was associated with the overall capability to adopt healthy lifestyles, particularly regarding overall CADA and physical activity scores. Individuals with low or middle income demonstrated lower scores than their wealthier counterparts. Alageel et al. suggested that behavior change interventions for individuals with limited resources should address barriers and improve the affordability and accessibility of healthy options to reduce health disparities (24). Housing type also played a significant role, with residents of family houses and duplexes reporting higher overall CADA, physical activity, and diet scores than those living in apartments or other housing types. This association could be linked to lifestyle differences associated with living space, family structure, and access to recreational areas or facilities conducive to physical activity. Similar to our findings, a study among patients with diabetes in Saudi Arabia demonstrated a link between housing type and the lifestyle choices of the participants (25). Another study conducted among adolescents in Italy showed that environmental choice, proximity to green areas, and family impact the chance of adopting a healthy lifestyle (26).

The geographical region also remained an independent, influential factor; notably, residents in central regions showed greater capabilities to adopt healthy lifestyles than those in other regions, as cultural factors may influence lifestyle choices. Overweight individuals demonstrate better capability or engagement in adopting healthier behaviors across various lifestyle domains, including dietary choices. In contrast, underweight individuals and those with a normal weight did not significantly differ from extremely obese individuals in their capabilities to adopt a healthy lifestyle. Both physical and psychological perceived health strongly influence individuals’ capability to adopt healthy lifestyles. Participants reporting no perceived physical limitations or psychological concerns consistently demonstrated higher scores across overall CADA, physical activity, and diet. This underscores the critical role of subjective health perceptions in shaping health behaviors, suggesting that interventions promoting positive health perceptions could enhance lifestyle behaviors. Similar to findings from a study conducted in Korea, there is a notable correlation between dietary habits and perceived physical and mental health; however, the cross-sectional design precludes determining causality (27). In contrast, the presence of diagnosed chronic physical or psychological conditions did not significantly influence the scores for the capabilities to adopt healthy lifestyles. This finding suggests that while perceived health status is pivotal, diagnosed health conditions may not always directly impact individuals’ engagement in health-promoting behaviors. Future studies could further explore the relationships among health conditions, perceived health, and actual health behaviors.

The findings of this study offer valuable insights that can shape public health policies and strategies to promote healthier lifestyles. The strong associations between socioeconomic factors, housing type, and perceived health status with lifestyle behaviors suggest that public health interventions should be tailored to address these differences. Targeted health promotion initiatives that address differences could also be beneficial (28). Moreover, improving public health literacy on the role of mental and physical well-being in adopting healthy behaviors is essential. Interventions promoting positive health perceptions, especially in communities with low perceived health status, could motivate individuals to adopt healthier lifestyles. This approach would align with a broader strategy of promoting holistic well-being, emphasizing physical and mental wellness. Consistency with previous studies by Alenzi showed that males were less likely to look at the labeled calorie information in restaurant menus (27); addressing gender-specific needs, such as tailoring nutritional education to male populations, who tend to exhibit lower diet scores, could help bridge the gap in diet-related health behaviors. Finally, these findings underscore the importance of designing interventions integrating physical activity and dietary components rather than treating them as separate domains. By doing so, public health strategies can create a more cohesive and sustainable approach to improving lifestyle behaviors across different population groups.

The current study possesses strengths and limitations that must be considered. To our knowledge, this is the first study to evaluate the capacity for adopting a healthy lifestyle among a large Arabic sample in Saudi Arabia, utilizing a comprehensive sociodemographic dataset. However, several limitations must be acknowledged when interpreting the results. First, using a cross-sectional study design may restrict the ability to establish causal relationships. Therefore, future research employing longitudinal designs would be beneficial to explore causal inferences over time. Second, the convenience sampling technique may not fully represent the broader population, potentially impacting the generalizability of the findings. Thus, using random sampling methods in upcoming studies will enhance the sample’s representativeness and improve external validity. Third, the self-reported data on diagnosed diseases, weight, and height could be subject to recall bias, further influencing the accuracy of the findings. Another limitation of this study is its focus on only two components of a healthy lifestyle, diet and physical activity, excluding other potentially important factors such as sleep and stress management. Therefore, future research should aim to incorporate a broader range of lifestyle behaviors to provide a more holistic understanding of their effects on health outcomes. Lastly, future research could explore additional factors, such as environmental and cultural influences, that may contribute to adopting healthy lifestyles. Studies could also further explore the specific impact of certain types of chronic diseases on the ability to adopt healthy lifestyles. A deeper exploration of these variables could provide a more comprehensive understanding of the factors influencing lifestyle behaviors in diverse populations. Future studies can expand upon the findings by addressing these limitations and offering more robust conclusions.

In conclusion, this study offers valuable insights into the multifaceted determinants of healthy lifestyle adoption derived from both bivariate and multivariate analyses. The findings reveal strong associations between socioeconomic factors, housing type, weight status, and perceived health status with lifestyle behaviors. Specifically, age and education were identified as significant factors that may mediate other variables that influence the ability to adopt healthier lifestyles. Conversely, gender, income, type of housing, weight status, and perceived physical and psychological health emerged as key drivers of health behaviors. These findings emphasize the necessity of addressing socioeconomic disparities, gender differences, housing environments, regional variations, and subjective health perceptions in the design of targeted public health interventions. By tailoring strategies to address these factors, public health efforts can promote healthier lifestyles across diverse populations more effectively. This comprehensive understanding will help refine approaches to enhance health outcomes at the population level.

## Data Availability

The original contributions presented in the study are included in the article/supplementary material, further inquiries can be directed to the corresponding author.
